# Pharmacological and Clinical Significance of Heme Oxygenase-1

**DOI:** 10.3390/antiox10060854

**Published:** 2021-05-27

**Authors:** David E. Stec, Nader G. Abraham

**Affiliations:** 1 Department of Physiology and Biophysics, Cardiorenal and Metabolic Diseases Research Center, University of Mississippi Medical Center, Jackson, MS 39216, USA; 2 Department of Medicine, New York Medical College, Valhalla, NY 10595, USA; 3 Department of Pharmacology, New York Medical College, Valhalla, NY 10595, USA; 4 Joan C. Edwards School of Medicine, Marshall University, Huntington, WV 25701, USA

This Special Issue collates and updates the current knowledge of the pharmacology and clinical applications concerning the enzyme heme oxygenase (HO). Heme oxygenases comprising of HO-1 (inducible isoform) and HO-2 (constitutive isoform) break down heme to equimolar amounts of the bile pigment biliverdin, carbon monoxide (CO), and iron (Figure 1). Heme oxygenases catalyze heme degradation using electrons supplied by NADPH–cytochrome P450 oxidoreductase (CPR). HO-1 is induced as an intracellular defense system that affords cytoprotection against numerous pathological conditions. HO-2 is the constitutive isoform that is critical to cellular and vascular homeostasis. Biliverdin is then reduced to bilirubin by the ubiquitous enzyme biliverdin reductase. Bilirubin is one of the most potent antioxidants in the body, and also has anti-inflammatory properties (Figure 1). CO is also an important anti-inflammatory agent through its suppression of cytokines. It can also stimulate guanylate cyclase, increasing cyclic guanosine monophosphate (cGMP) levels and promoting vasodilation of the vasculature (Figure 1). The breakdown of heme by HO enzymes is vital to maintain normal cellular function. Free heme stimulates increases in cellular reactive oxygen species generation and inflammation (Figure 1). The HO-1 pathway is crucial in cellular defense for numerous diseases, including diabetes, hypertension, heart diseases, inflammation, transplantation, neurodegeneration, aging, cancer, and COVID-19 [1,2,3,4,5].

This volume covers the background and essential role of HO in normal physiology and disease. In the past 10–15 years, an emphasis on the genetic manipulation and development of novel specific HO inducers and inhibitors to alter HO activity and the levels of its metabolites, CO, and bilirubin has increased the knowledge of the HO pathway in physiological and pathological conditions. Classically, HO-1 is observed as a 32 kDa protein, found abundantly in the microsomal fraction due to its association with the Endoplasmic Reticulum (ER) [6]. However, it localizes to the cytoplasm as a ~14 kDa fragment and a ~28 kDa protein in the nucleus and mitochondria, respectively [7]. Several pathological conditions such as Alzheimer’s, preeclampsia, and kidney injury exhibit increased plasma and urinary levels of HO-1, and increased tissue levels of HO-1 could be a biomarker for these diseases [8,9,10]. Several conditions such as diabetes, hypertension, and obesity are associated with deficiencies or reductions in HO-1 [6]; however, increased HO-1 levels can also be detrimental, especially in cancer [11].

The therapeutic potential of the HO pathway is evident from the many different ways it is being manipulated to treat a wide variety of diseases. Therapeutic strategies ranging from genetic approaches to increase HO-1 protein to chemical or natural product HO-1 inducers, have found promise for cardiovascular, metabolic, and inflammatory diseases [12,13]. At the same time, blockades of HO-1 activity through the use of genetic approaches or specific HO inhibitors have increased our knowledge of the physiological role of this system, and provided novel therapeutics for diseases such as neonatal jaundice [14].

The expertise of the Editors of this Special Issue is in the biochemistry, pharmacology, and physiology of HO-1, and its products in cardiovascular and metabolic diseases. Dr. Abraham’s group has developed novel methods of overexpression of HO-1 using lentiviral vectors to determine the role of HO-1 in hypertension, diabetes, and obesity [15,16]. Dr. Stec’s group has utilized novel mouse models of HO-1 and biliverdin reductase-A (BVRA) to determine the role of HO-1 and bilirubin in kidney function, obesity, diabetes, and non-alcoholic fatty liver disease (NAFLD) [17,18,19]. The vast scope of the HO system in a wide variety of pathological conditions is evident from the manuscripts comprising this Special Issue. From well-established diseases such as cancer, hypertension, diabetes, and Alzheimer’s disease, to newly emerging pandemics such as COVID-19, elevated levels of HO-1, and heme degradation, products play an important role in these diseases’ underlying physiology and offer new potential therapeutic approaches for their treatment. Prominent scientists listed in this volume have made immense contributions to clarify the role of the HO-1 pathway in several areas. They have also expanded the translation of this knowledge to clinical research. We believe that this Special Issue reflects the diverse roles that the HO system plays in many different diseases, and highlights the most innovative research. We want to extend our most profound appreciation to all of the authors who contributed their work to this Special Issue.

## Figures and Tables

**Figure 1:**
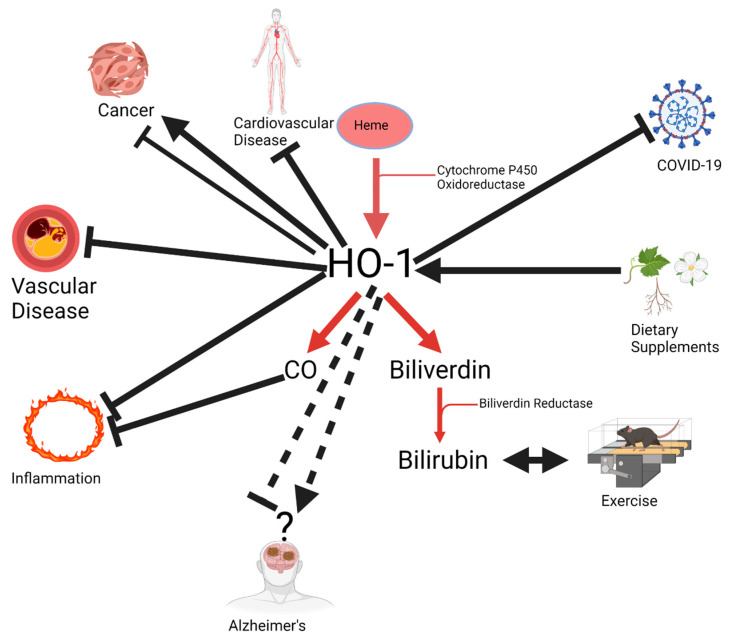
Pleiotropic effects of heme oxygenase-1 (HO-1). HO-1 has both stimulatory and inhibitory effects on cancer. It inhibits vascular disease, inflammation, COVID-19, and cardiovascular disease. It can be upregulated by a wide variety of dietary supplements. Enhancement of HO-1 activity increases carbon monoxide, which is a potent anti-inflammatory molecule, and increases the levels of bilirubin which are also augmented by exercise. Arrows indicate increased activity, lines with bars indicate inhibitory actions, and dashed lines indicate unknown function. Figure created with Biorender.com.
